# Gemcitabine as chemotherapy of head and neck cancer in Fanconi anemia patients

**DOI:** 10.1038/s41389-024-00525-2

**Published:** 2024-07-11

**Authors:** Anne M. van Harten, Ronak Shah, D. Vicky de Boer, Marijke Buijze, Maaike Kreft, Ji-Ying Song, Lisa M. Zürcher, Heinz Jacobs, Ruud H. Brakenhoff

**Affiliations:** 1 Amsterdam UMC location Vrije Universiteit Amsterdam, Otolaryngology-Head and Neck Surgery, Head and Neck Cancer Biology & Immunology Section, De Boelelaan 1117, 1081 HV Amsterdam, The Netherlands; 2 Cancer Center Amsterdam, Cancer Biology and Immunology, Amsterdam, The Netherlands; 3 Division of Tumor Biology & Immunology, The Netherlands Cancer Institute, Amsterdam, The Netherlands; 4 Department of Experimental Animal Pathology, The Netherlands Cancer Institute, Amsterdam, The Netherlands

**Keywords:** Head and neck cancer, Target identification, Drug safety, Cancer genetics, Target validation

## Abstract

Fanconi anemia (FA) is a rare hereditary disease resulting from an inactivating mutation in the FA/BRCA pathway, critical for the effective repair of DNA interstrand crosslinks (ICLs). The disease is characterized by congenital abnormalities, progressing bone marrow failure, and an increased risk of developing malignancies early in life, in particular head and neck squamous cell carcinoma (HNSCC). While ICL-inducing cisplatin combined with radiotherapy is a mainstay of HNSCC treatment, cisplatin is contra-indicated for FA-HNSCC patients. This dilemma necessitates the identification of novel treatment modalities tolerated by FA-HNSCC patients. To identify druggable targets, an siRNA-based genetic screen was previously performed in HNSCC-derived cell lines from FA and non-FA tumor origin. Here, we report that the Ribonucleotide Reductase (RNR) complex, consisting of the RRM1 and RRM2 subunits, was identified as a therapeutic target for both, FA and non-FA HNSCC. While non-FA HNSCC cells responded differentially to RNR depletion, FA-HNSCC cells were consistently found hypersensitive. This insight was confirmed pharmacologically using 2′, 2′-difluoro 2′deoxycytidine (dFdC), also known as gemcitabine, a clinically used nucleotide analog that is a potent inhibitor of the RNR complex. Importantly, while cisplatin exposure displayed severe, long-lasting toxicity on the hematopoietic stem and progenitor compartments in *Fancg−/−* mice, gemcitabine was well tolerated and had only a mild, transient impact. Taken together, our data implicate that gemcitabine-based chemoradiotherapy could serve as an alternative HNSCC treatment in Fanconi patients, and deserves clinical testing.

## Introduction

Fanconi anemia (FA) is a rare genetic disorder caused by a mutation in one of the 22 Fanconi anemia genes, leading to defective FA/BRCA interstrand crosslink (ICL) repair pathway, hampering successful removal of ICLs during and after DNA replication [1, 2]. Patients with FA are characterized by congenital abnormalities, bone marrow failure, a shortened life expectancy, and hypersensitivity to ICL agents, such as mitomycin C and DNA platinating agents, including the widely applied cytotoxic drug cisplatin [3]. Furthermore, FA patients are predisposed to develop malignancies early in life, including a 500- to 800-fold increased risk for head and neck squamous cell carcinoma (HNSCC) compared to the general population, with most tumors arising in the oral cavity. The median onset age of HNSCC in FA patients is 30 years [4–6], while in non-FA patients this is generally around 60–70 years of age. In non-FA patients, risk factors for HNSCC are either carcinogen exposure from tobacco and excessive alcohol consumption, or a persistent infection with a high-risk type of the human papillomavirus (hrHPV). In contrast, cancers in FA patients may also arise independently of exogenous carcinogen exposure. The DNA-repair defect causes the accumulation of highly mutagenic ICLs by endogenous metabolites like aldehydes instigating the onset of neoplasms [7–9].

Treatment for early-stage HNSCC consists of surgery or radiotherapy. Advanced stages of disease outside the oral cavity are treated by cisplatin-based chemotherapy concomitantly with locoregional radiotherapy (chemoradiotherapy). For advanced-stage oral cancers surgery is applied, followed by postoperative radiotherapy or chemoradiotherapy [7]. For patients unfit to receive cisplatin, anti-EGFR (cetuximab) can be applied, which is less effective [10]. A germ-line FA defect has profound consequences regarding the treatment of HNSCC. Due to severe toxicity, FA patients with HNSCC cannot sustain cisplatin, and therefore, the clinical management is challenging with treatment options being limited to surgery and/or radiotherapy, and consequently, survival rates are disappointing [11–13]. Hence, novel treatment protocols are urgently required to treat FA-HNSCC. Drugs require proper preclinical testing in FA-deficient mouse models to test the expected tolerability in FA patients and exclude on beforehand unforeseen toxicities that may be severe.

Previously, we reported on a panel of 319 tumor-lethal siRNAs that emerged from genome-wide screens in cancer cells. These were rescreened on a large panel of HNSCC cell lines including FA-HNSCC derived cell lines [14]. Here, we aim to identify druggable gene targets that could be exploited for treatment of FA-HNSCC and we provide preclinical data on gemcitabine toxicity in FA-HNSCC cell lines, FA-deficient cells and FA-deficient mice.

## Results

### *RRM1* and *RRM2* are essential genes in FA-HNSCC

**Fig. 1:**
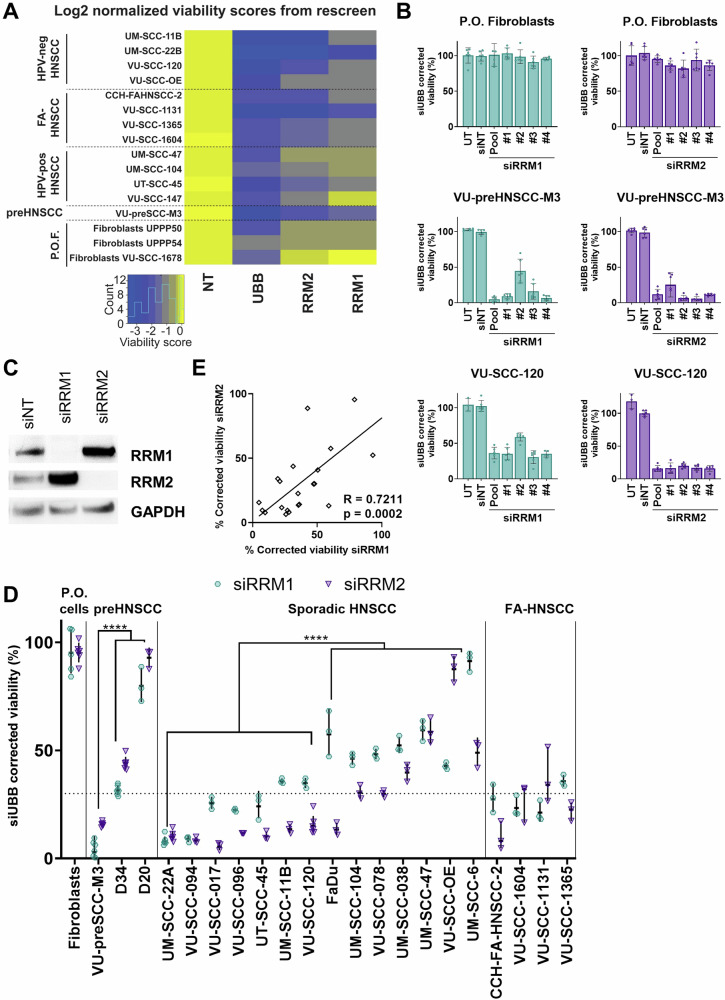
*RRM1* and *RRM2* genes are essential in FA-HNSCC cell lines. **A** Heatmap of Log2 normalized viability scores obtained by a siRNA library rescreen in a panel of Fanconi anemia-derived HNSCC, HPV-negative and HPV-positive HNSCC, premalignant oral cells, and primary oral fibroblasts (P.O.F.). Note that fibroblasts VU-SCC-1678 are primary fibroblasts that emerged when VU-SCC-1678 was cultured, and these should be considered as cancer-associated fibroblasts. All experiments were performed in triplicate. NT non-targeting siRNA control, *UBB* viability score obtained after knockdown of *Ubiquitin B* (*UBB*) as positive transfection control. **B** Viability after mRNA knockdown of *RRM1* (turquoise) and *RRM2* (purple) by the pooled siRNA and the subsequent individual siRNAs (#1–#4) in primary oral fibroblasts, premalignant oral cells (VU-preSCC-M3) and HPV-negative cell line VU-SCC-120. Viability was normalized to siNT and corrected for the transfection efficiency determined by si*UBB*. All experiments were performed in triplicate. **C** Assessment of protein expression of RRM1 and RRM2, 48 h post-transfection with pooled siRNAs, indicates (near) complete knockdown. Increased protein levels of RRM2 are observed when RRM1 is knocked-down and vice versa. **D** Summary of the viability after *RRM1* and *RRM2* knockdown for all cell lines tested. All tested FA-HNSCC cell lines showed vulnerability to *RRM1* and *RRM2* knockdown, whereas for the non-FA HNSCC cell lines 7/14 showed sensitivity and 1/3 of the premalignant cells. The tested primary oral fibroblast was resistant to RNR complex knockdown. ****p < 0.0001 with *t*-test statistic. **E** The effects on viability upon knockdown of either RNR complex gene, highly correlates for all cell lines tested (Pearson’s correlation *R* = 0.7211, *p* = 0.0002).

As a starting point of this study, we analyzed the viability data of all cell lines from the previously executed 319 tumor-lethal siRNA re-screen [14], focusing specifically on the FA-HNSCC cell lines. A viability score of ≤−0.5 relative to the positive and negative controls was considered lethal [14] (Fig. 1a). We noted that the knockdown of both *RRM1* and *RRM2* resulted in a highly significant reduction of viability, which was observed in all FA-derived and the majority of non-FA-derived cell lines tested.

Ribonucleotide reductase catalytic subunits M1 and M2, RRM1 and RRM2, respectively, are key components of the ribonucleotide reductase (RNR) complex. *RRM1* encodes the ribonucleoside-diphosphate reductase large subunit that forms the α-subunit of the holoenzyme ribonucleotide reductase (RNR) complex. Two proteins may function as β-subunit in the complex. Primarily RRM2 forms a functional heterodimeric tetramer with RRM1, but its paralogue ribonucleotide-diphosphate reductase subunit M2 B (RRM2B, formerly known as p53R2) can complex with RRM1 as well to form a functional RNR enzyme [15]. The RNR complex catalyzes de novo production of deoxyribonucleoside diphosphates and triphosphates (dN(D)TPs) from ribonucleoside di- and triphosphates (N(D)TPs) to provide proliferating cells with the required deoxynucleotides for DNA replication in S-phase. In quiescent cells, dNTPs are also generated, but particularly for DNA repair [15].

To investigate the susceptibility of nonmalignant cells, we analyzed the viability score after *RRM1* and *RRM2* knockdown in primary non-transformed oral (PO) fibroblasts from two healthy donors (donors #50 and #54) and from an FA patient (VU-1678). The normal fibroblasts did not reach the lethality threshold, indicating a possible therapeutic index to target HNSCC cells also of FA patients.

Next, to evaluate the effects on cell viability and to exclude off-target effects, the four individual siRNAs that are present in the SMARTpools, and used as such in the initial screen and re-screens, were individually transfected into PO fibroblasts and several cell lines. All four individual siRNAs targeting *RRM1* or *RRM2* caused a reduced viability in the tumor cell lines but not in the normal PO fibroblasts (Figs. 1b and S1a).

Protein expression of RRM1 and RRM2 was assessed 48 h post-transfection with the SMARTpools and indicated a large reduction of protein levels. In addition, the knockdown of one counterpart caused upregulation of the other one, consistent with previous notions [16] (Fig. 1c).

A larger panel of cell lines was analyzed for *RRM1* and *RRM2* knockdown, including FA-HNSCC cell lines CCH-FAHNSCC-2, VU-SCC-1604, VU-SCC-1131, and VU-SCC-1365. All four tested FA lines were below a relative viability of 0.3; an arbitrary cut-off that separates sensitive from resistant cell lines. (Fig. 1d and S1b–e, Table S1). The observed effect on viability to *RRM1* knockdown correlated significantly to effects obtained with *RRM2* knockdown (Pearson’s correlation test, *R* = 0.67, *p* = 0.0008) (Fig. 1e). Nonetheless, variations were observed; some non-FA HNSCC cell lines displayed almost identical sensitivity to *RRM1* and *RRM2* knockdown (e.g., UM-SCC-22A), while others (e.g., UM-SCC-6) displayed a considerable difference (Fig. 1d).

To investigate whether the cell lines could be further sensitized to RNR complex interference, we combined the knockdown of *RRM1* and *RRM2* in a dual transfection (Fig. S1b–e). The combination did not enhance the lethal effects, neither in the more sensitive nor the more resistant tumor cell lines.

### Response of FA-HNSCC cell lines to RNR inhibitor gemcitabine

Gemcitabine or 2′, 2′-difluoro-2-deoxycytidine (dFdC) is a deoxycytidine analog and a known inhibitor of the RNR complex. After uptake, dFdC is phosphorylated to monophosphate dFdCMP [17]. Further phosphorylation steps lead to the formation of dFdCDP and dFdCTP, of which the first is a potent inhibitor of the RNR complex and the latter is known to be incorporated into the DNA [18, 19]. Gemcitabine is a clinically approved drug and established as therapy for e.g., pancreatic cancer and non-small-cell lung cancer, but not for HNSCC [18, 20, 21].

**Fig. 2:**
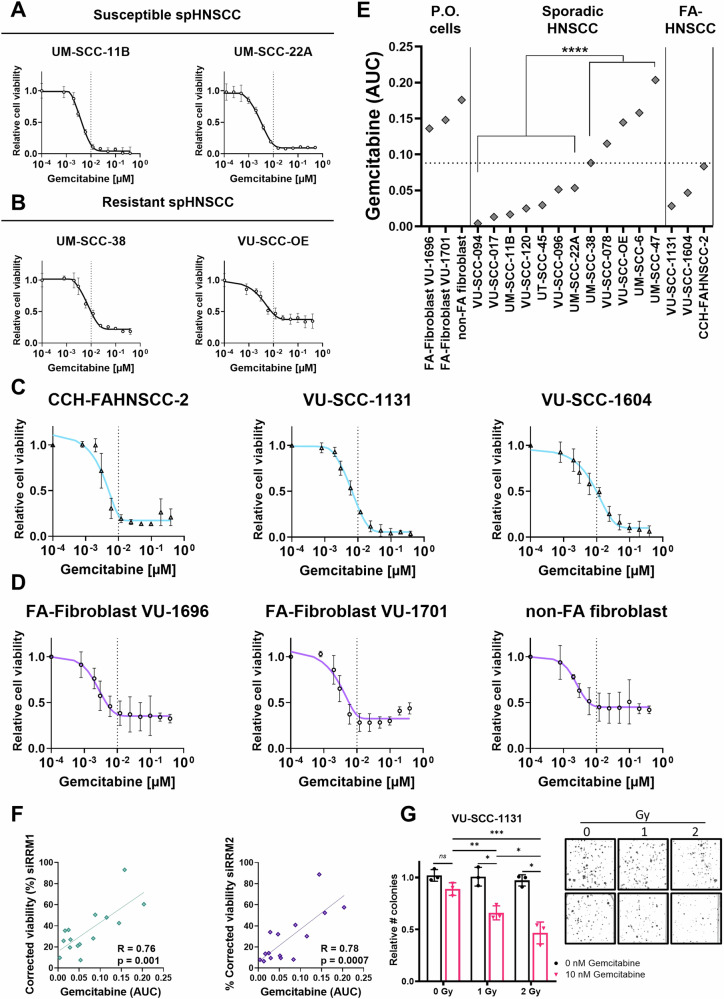
Ribonuclease reductase (RNR) complex inhibition through gemcitabine shows tumor-specific vulnerability in Fanconi anemia-derived FA-HNSCC cell lines. **A** Viability was obtained after 72 h of treatment with a dose titration of gemcitabine in susceptible sporadic non-FA HNSCC (spHNSCC) and **B** resistant spHNSCC cell lines. The dashed line indicates an arbitrary threshold of 0.01 µM gemcitabine as a visual reference. All experiments in **A**–**D** were performed three times in triplicate. **C** Viability of three independent Fanconi anemia HNSCC cell lines after gemcitabine treatment. All FA-HNSCC cell lines showed vulnerability to gemcitabine treatment. **D** Viability of primary fibroblasts from three independent donors, of which two from Fanconi anemia patients, were all resistant to gemcitabine treatment. **E** Summary of all viability data after gemcitabine exposure. The area under the curve (AUC) is shown. With an AUC threshold of 0.08, all cell lines are equally sensitive to gemcitabine as to RNR knockdown. **F** Gemcitabine susceptibility significantly correlates with susceptibility to RNR complex knockdown (Pearson’s correlation gemcitabine (AUC) to si*RRM1* *R* = 0.76, *p* = 0.001; Pearson’s correlation gemcitabine (AUC) to si*RRM2* *R* = 0.78, *p* = 0.0007). **G** Colony formation assay assessing the synergy between gemcitabine and radiation, indicated an dose–response significant response. Representative pictures of colonies are shown. *p ≤ 0.05, **p ≤ 0.01, ***p ≤ 0.001, **** ≤ 0.0001 with Student’s *t*-test.

Therefore, we proceeded with testing gemcitabine sensitivity for FA-HNSCC (Fig. 2a–e and S2). Data were compared by AUC analysis on the dose-response curves as more resistant cell lines did not show a major shift in the curve, but just did not reach a complete viability reduction at higher concentrations of gemcitabine, hampering a reliable EC_50_ concentration calculation. A panel of FA- and non-FA-HNSCC cell lines and PO fibroblasts from two FA patients and one healthy donor were treated with a dose range of gemcitabine. Cell lines such as VU-SCC-OE that were more resistant to siRNA knockdown (Fig. 1), were also more resistant to gemcitabine treatment. The FA-HNSCC cell lines were all sensitive to gemcitabine treatment (Fig. 2c), in line with the RNA interference data of *RRM1* and *RRM2*. Notably, both the FA- and non-FA fibroblasts were relatively resistant to gemcitabine (Fig. 2d), excluding a synthetic lethal interaction between RNR inhibition and a defective FA/BRCA pathway.

Sensitivity to gemcitabine highly correlated with *RRM1* and *RRM2* knockdown, highlighting the specific targeting of gemcitabine as an RNR complex inhibitor (Pearson’s correlation test, *RRM1*-AUC, *R* = 0.75, *p* = 0.001; *RRM2*-AUC *R* = 0.89, *p* < 0.0001) (Fig. 2f).

As we expect that gemcitabine will be combined with radiotherapy in clinical setting, we tested the additive effect of both treatments in FA-HNSCC cell line VU-SCC-1131. A synergistic dose-responsive anti-tumor effect was observed in a clonogenic assay when cells were treated with 10 nM gemcitabine and exposed to 0, 1, or 2 Gray. (Fig. 2g).

To analyze gemcitabine and radiation sensitivity in 3D organoid cultures, both VU-SCC-1131 and VU-SCC-1604 were exposed to gemcitabine 24 h and 5 days after seeding both in 2D and 3D. Macroscopic and microscopic pictures of organoid cultures are indicated in Suppl Fig. S3a, b. Gemcitabine was added after either 24 h (2Ds, 3Ds) or after 5 days (2De, 3De). Whether cells were cultured in 2D or 3D did not differ with respect to gemcitabine response. However, longer preincubation culturing periods and formation of tumor-like structures caused an increase of the EC_50_ for both 2D and 3D with approximately 10× (VU-SCC-1604) or 20× (VU-SCC-1131). Also for irradiation, 2D or 3D culturing did not impact radiation sensitivity.

### Hematopoietic analysis in *Fancg−/−* mice exposed to gemcitabine

**Fig. 3:**
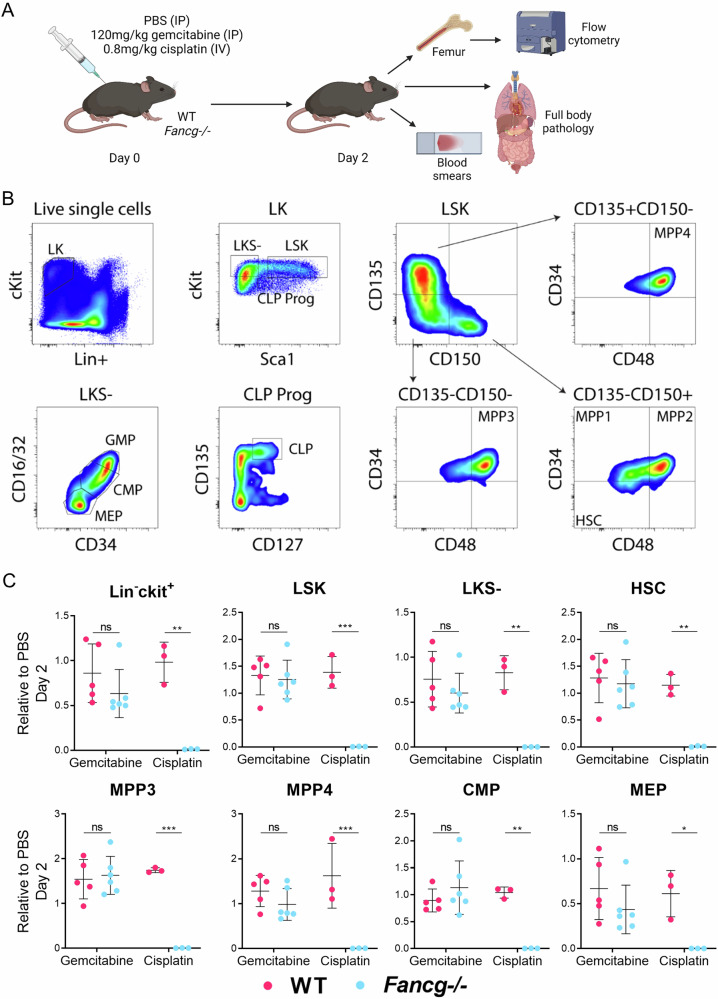
Cisplatin but not gemcitabine exposure affects hematopoietic subsets in *Fancg−/*− mice. **A** Schematic outline of the toxicity study. Figure prepared using BioRender. **B** Flow cytometry-based gating strategy to identify different hematopoietic cell subsets. Bone marrows were flushed, erylyzed, and stained with a cocktail of surface antigen markers, and live single cells were used to distinguish the different subsets. **C** Relative numbers of different subsets as defined in (**B**), analyzed on day 2 post treatment. The graphs indicate the fold change of cells relative to PBS controls. In each graph, the bar represents mean ± sd. WT gemcitabine (*n* = 5), *Fancg−/−* gemcitabine (*n* = 6), WT cisplatin (*n* = 3) and *Fancg−/−* cisplatin (*n* = 3). *p* Values were calculated using two-way ANOVA. *p = 0.05, **p < 0.01, ***p < 0.001, ****p < 0.0001.

The main concern when treating FA patients with chemotherapeutic agents is toxicity. Both toxicity in general and, most notably bone marrow failure due to the hypersensitivity of hematopoietic stem and progenitor cells (HSPCs) to genotoxic agents [22, 23]. To study the impact of gemcitabine in vivo, wild-type (WT) and *Fancg* null (*Fancg−/−*) mice were either treated with PBS, 120 mg/kg gemcitabine [24], or 0.8 mg/kg cisplatin (positive control). Bone marrow (BM) was harvested two days later for downstream analysis (Fig. 3a). Using an established panel of hematopoietic stem and progenitor cell (HSPC) markers [25], multiplex flow cytometry was performed to identify and analyze different HSPC populations (Fig. 3b). In mice, HSPCs reside within the LSK compartment of bone marrow cells, defined as Lineage (Lin)-negative cells expressing high levels of the Stem cell antigen-1 (Sca-1) and c-Kit receptor. For cisplatin, in comparison to WT mice, the LSK population of *Fancg−/−* mice was found hypersensitive resulting in a near ablation of HSPCs (Fig. 3c, Figure S4a). In contrast, gemcitabine-treated HSPCs displayed no reduction in both WT and *Fancg−/−* mice. Apart from stem and multipotent progenitors (MPPs), myeloid-committed cells such as common myeloid progenitors (CMP) and megakaryocyte–erythroid progenitors (MEP), were also strongly affected in cisplatin-treated *Fancg−/−* mice (Fig. 3c, Figure S4a). This illustrates that a relatively low dose of cisplatin leads to severe toxicity in the entire hematopoietic network of *Fancg−/−* mice, strongly affecting the survival and renewal of short-term and long-term cells that warrant blood homeostasis. In contrast to cisplatin, CMPs and MEPs in *Fancg−/−* mice were also hardly affected by gemcitabine. In summary, a clinically relevant dose of gemcitabine renders no toxicity to the HSPCs in FA mice, whereas a relatively low dose of cisplatin had a major impact as expected. The data in these mouse models suggest that gemcitabine may be utilized as a potential therapeutic agent in FA patients without causing additional toxicity by the FA defect.

### Histopathological analysis of gemcitabine-treated *Fancg−/−* mice

**Fig. 4:**
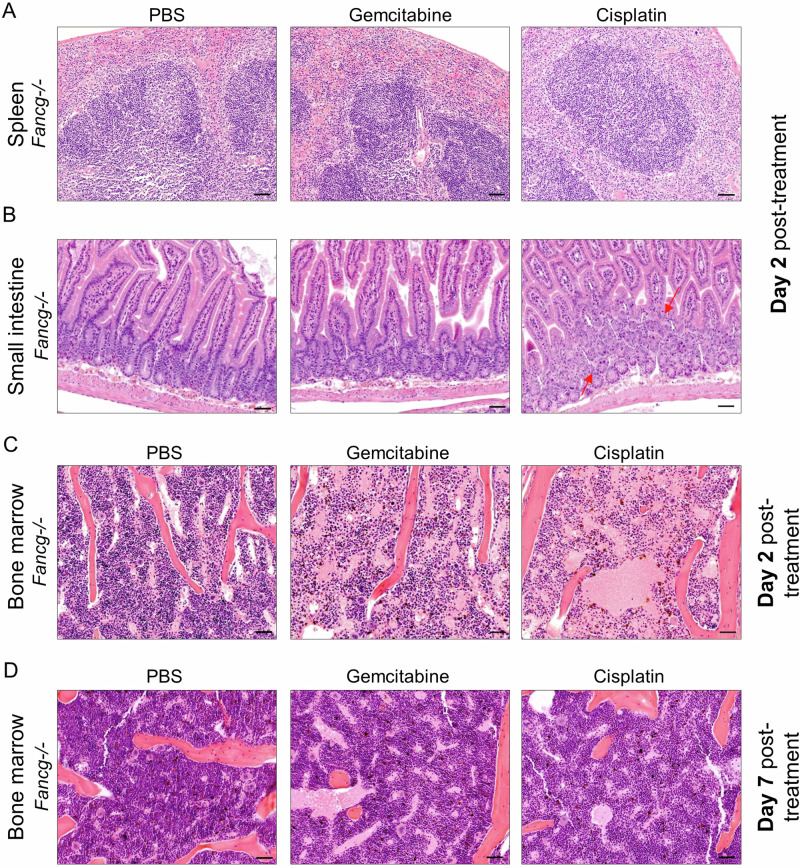
Gemcitabine is well tolerated by *Fancg−/−* mice with a transient effect on bone marrow cellularity. **A**–**C** Representative H&E sections of the spleen (**A**), small intestine (SI) (**B**), and bone marrow (BM) (sternum) (**C**) isolated from *Fancg−/−* mice treated with PBS, 120 mg/kg gemcitabine or low dose 0.8 mg/kg cisplatin and harvested on day 2 post-treatment. Red arrows indicate apoptotic cells. **D** Representative H&E sections of BM (sternum) isolated from *Fancg−/−* mice treated with PBS, 120 mg/kg gemcitabine, or 0.8 mg/kg cisplatin and harvested on day 7 post-treatment.

To identify if gemcitabine exposure induces any other tissue toxicity in *Fancg−/−* mice, a whole-body pathology examination was performed on mice from all genetic backgrounds and treatment groups. Histopathological analysis was performed blinded on blood smears, spleen, thymus, heart, lung, reproductive organs, BM and small intestine (SI), to examen systemic toxicities. Giemsa-Wright staining of blood smears and H&E staining of the spleen (Fig. 4a), thymus (Figure S5a), heart, lung, and reproductive organs (not shown) did not reveal any gross pathological alterations upon gemcitabine- or cisplatin-exposure of *Fancg−/−* mice in comparison to PBS controls. In contrast to these tissues, the SI of *Fancg−/−* mice was not impacted by gemcitabine treatment but considerably impacted by cisplatin exposure, as indicated by the disrupted tissue and absence of defined crypts (Fig. 4b). The absence of a functional FA/BRCA pathway and the rapid cell turnover makes the SI also hypersensitive to cisplatin in the mice, but not to gemcitabine exposure in line with the bone marrow data (Fig. 3).

Although hematopoiesis was not affected in *Fancg−/−* mice 48 h post-gemcitabine treatment (Fig. 3c), mild toxicity in cellularity was observed in the H&E slides of the bone marrow (Fig. 4c). This was categorized as a mild effect as gemcitabine treatment decreased the absolute cell numbers, but the proportion of each hematopoietic subset was not affected. On the contrary, the cisplatin-treated *Fancg−/−* mice experienced severe toxicity, as both total cellularity (H&E slide) (Fig. 4c) as well as proportion of each subset was reduced substantially as determined by flow cytometry (Fig. 3c).

Gemcitabine is generally administered on a weekly basis in human clinical trials [26]. Therefore, we questioned whether the subtle reduction in BM cellularity on day two in *Fancg−/−* mice was restored by day seven. To test this hypothesis, mice experiments were repeated as described above, and the histopathology and flow cytometry analysis was performed on day seven post-treatment. In line with our hypothesis, H&E sections of the BM from gemcitabine-treated *Fancg−/−* mice showed a complete recovery (Fig. 4d). Interestingly, cisplatin-treated SI and BM analysis unexpectedly suggested a complete tissue recovery, based on the presence of nucleated cells (Figure S5b). However, flow cytometry data revealed that the hematopoietic subsets in cisplatin-treated *Fancg−/−* mice remained significantly reduced on day seven (Fig. S5c), as expected in FA mice and, by extension in FA patients. After treatment with gemcitabine, cell populations were restored at day 7, which again confirms that contrary to cisplatin, a deficient FA/BRCA pathway is not synthetically lethal with gemcitabine treatment.

In summary, our data strongly indicates that treatment with gemcitabine is well tolerated by FA/BRCA deficient mice and may provide an alternative treatment modality for chemoradiotherapy of HNSCC in FA patients.

## Discussion

Given the central role of the FA pathway in ICL repair, FA patients with germline mutations in one of the 22 FA genes are defective in DNA repair leading to a high tumor incidence. FA patients develop acute myelogenous leukemia (AML) with an incidence that is 700-fold higher compared to the general population [4, 27]. FA patients who overcome severe bone marrow failure following a successful bone marrow transplant are still likely to develop esophageal, gastrointestinal, vulvar, and, most particularly head and neck cancers [4]. The standard of care for HNSCC often includes systemic cisplatin, which causes severe toxicity or even lethality for the FA-patient group combined with concomitant radiotherapy [7, 8]. The addition of cisplatin improves 5-year survival by at least 6.5% in non-FA HNSCC patients [28, 29], and alternatives for cisplatin are less effective [30]. Hence, new chemoradiotherapy strategies are needed for FA patients, and in fact for all patients unfit to receive cisplatin, including elderly patients over 70 years who do not benefit from cisplatin [29]. Here, we show that the clinically approved drug gemcitabine may offer an interesting alternative for the treatment of FA patients with chemoradiotherapy. Our preclinical models indicated strong vulnerabilities of FA-HNSCC to gemcitabine, also in combination with radiation, while it is well-tolerated by HSPCs and other tissues of FA-deficient mice, certainly when compared to the devastating effects of low doses of cisplatin.

The nucleotide analog 2′, 2′-difluoro 2′deoxycytidine, also known as dFdC or gemcitabine, is a potent inhibitor of the RNR complex, an essential complex in FA- and non-FA HNSCC. Further phosphorylation steps lead to the formation of dFdCDP and dFdCTP, of which the first is a potent inhibitor of the RNR complex and the latter a potent DNA polymerase inhibitor upon incorporation of dFdC into the DNA [17, 31]. This dual mode of action of gemcitabine on replication fork progression, i.e., replisome stalling by inhibiting the RNR complex and causing dNTP shortage as well as causing DNA lesions by incorporating dFdC, can be quite effective in cancer therapy [18, 19]. Recently, it has been shown that the 11q13 amplification, as often found in HNSCC tumors, caused a genetic rewiring of the cells and dependency on Cyclin D1 for cell proliferation while normal keratinocytes depend on cyclin D2. This specific amplification led to both RRM dependency and triapine (RRM2 inhibitor) sensitivity. Hence, all tumors with 11q13 amplification and cyclinD1 dependency are likely also RRM-dependent and gemcitabine-sensitive [32]. Gemcitabine has been tested for many cancer indications including the combination with radiotherapy in HNSCC [33], and has been approved for treatment of, e.g., bladder and pancreatic cancer. This makes off-label application in clinical studies in FA patients a realistic option, since data from our FA-mouse models suggest a well-tolerable bone marrow and other tissue toxicity profile.

An alternative therapy for cisplatin is cetuximab, which is also approved for HNSCC in combination with radiotherapy. However, the effectiveness of cetuximab is questioned, certainly when applied in HPV-positive tumors [34, 35]. Cetuximab has been applied in FA patients, but was not very effective and is associated with grade 3–4 toxicity. Furthermore, off-label use of other approved anti-cancer drugs has been described in FA-HNSCC patients (reviewed in [5]), without major successes at present [5]. Since FA-HNSCC often presents at an advanced stage, the 5-year survival is poor (39%). Therefore, new treatment modalities are urgently awaited.

Novel immunotherapies such as anti-PD-1 inhibition are nowadays registered for recurrent/metastatic HNSCC but obviously, cause concerns in FA-HNSCC after hematopoietic stem cell transplant (SCT). Two FA patients without SCT have been treated with pembrolizumab and one patient with nivolumab, which was well-tolerated [5, 36, 37]. Numbers are small, and PD-1 immunotherapy is currently not used in patients at first line. Although approved for recurrent/metastatic disease, the response rates are relatively low (~15%) in non-FA HNSCC [38]. Efforts are being made to utilize anti-PD1 antibodies as neoadjuvant treatment to surgery to improve clinical outcomes such as in the IMCISION trial [39] and NeoNivo trial (in press). A potential key problem in treating FA-HNSCC patients with immune checkpoint inhibitors relates to SCT. Around 75% of FA patients receive a SCT early in life, i.e., generally before the onset of HNSCC. The immune suppression related to ICT may elicit a host versus graft reaction (as reviewed in [40]), which may limit the application of immunotherapy in a substantial fraction of FA patients.

Although further clinical testing is required, our data suggest that gemcitabine may provide an interesting alternative. In line with this notion, a case report by Dudek et al. reported a transplanted FA patient treated with gemcitabine for a squamous cell carcinoma of the right lung [41]. Low toxicity was observed in the treated FA patient, and more importantly, resulted in a decrease of tumor volume (clinical response), which encourages further investigation of gemcitabine treatment in FA patients diagnosed with HNSCC. Chemoradiotherapy can be applied as a definitive modality, but is also combined postoperatively with surgery when unfavorable characteristics are noted by histological examination of the surgical specimen, such as tumor-positive surgical margins, non-cohesive growth, and extra nodular extension. Our data hopefully initiates a revival of gemcitabine as a chemotherapeutic agent in head and neck cancer management, particularly for patients unfit to receive cisplatin.

## Materials/subjects and methods

### Cell culture and gemcitabine testing

Primary keratinocytes and fibroblasts were collected from anonymized residual tissue samples according to the guidelines of the Dutch Medical Scientific Societies (www.federa.org). Samples from FA patients were collected according to the study protocol approved by the Institutional Review Board of the VU University Medical Center (protocol VUmc2003-001), with the Informed Consent of the patients. Primary cell cultures were generated as previously described [42]. The precancerous cultures were generated and cultured as previously described by us and others [43–45]. All tumor cell lines were cultured as described before [46–48].

All cells were always mycoplasma negative and regularly checked (Mycoalert, Lonza, LT07-318) and continuously authenticated by visual inspection and genetic profiling on indication. Cell lines were cultured to a maximum of 4 months after thawing.

Gemcitabine was obtained from Merck (G6423), and added in a concentration range of 0.8–390 nM 24 h after seeding of the cells. Cell viability was determined by CellTiter-Blue (Promega G8080) after 72 h. For read-out, a Glomax microplate reader was used. Dose–response curves were tested three times in triplicate. EC_50_ and the area under the curves were determined by GraphPad version 8.

### Clonogenic assays

Cells were seeded at low density in T25 cell culture flasks, and after 24 h treated with 10 nM gemcitabine for 24 h followed by irradiation using a ^57^Co source. The cells were incubated for at least 9 days, fixed, and stained with crystal violet and colonies were counted. Only colonies with >50 cells were counted. Number of colonies with treatment were relative to the number of colonies without treatment.

### 3D organoid cultures

In total 300 VU-SCC-1131 or VU-SCC-1604 cells were added to 5 µl geltrex (Thermo Fischer Scientific). After solidifying, 100 µl DMEM/F12 medium was added and the cells incubated, as described previously. Dependent on the experimental design, 25 µl medium with gemcitabine was added after 24 h or after 5 days. Concentration ranges were as described above. Alternatively, cells were after 5 days irradiated using a ^57^Co source. Next, CellTiter-Glo (Promega) was added, and luminescence was measured by a Glomax microplate reader (Promega) as indicated by the manufacturer. All experiments were performed in triplicate.

### siRNA transfection and deconvolution

siRNA transfections were performed in a 96-well plate (Greiner-Bio One, 655073) as previously described [14, 49]. As negative and positive transfection controls, siCONTROL#2 and si*UBB* (Ubiquitin B) were used, respectively. Cell viability was determined using CellTiter-Blue (Promega, G8080) after 96 h of treatment. Deconvolution was performed with both the siRNA SMARTpools and individual siRNA sequences targeting *RRM1* and *RRM2* (Dharmacon, Table S2). Transfections for *RRM2B* knockdown were performed with the siRNA SMARTpool only (Dharmacon, Table S2).

### Western blot analysis

Cells were seeded in a 6-well plate (Greiner-Bio One, 657160) and transfected with the siRNA SMARTpools. Forty-eight hours post-transfection, protein lysates were obtained using RIPA lysis and extraction buffer (Thermo Scientific, 89901) containing 1× HALT™ EDTA-free protease and phosphatase inhibitor cocktail (Thermo Scientific, 78447). Protein concentration normalized lysates (Pierce BCA Protein Assay Kit, Thermo Scientific, 23227) were run on 4–12% pre-casted SDS-PAGE gels (Bolt Bis-Tris Plus gels, Thermo Fisher, NW04122BOX) and blots developed after antibody incubation using Uvitec 47 Alliance reader (Uvitec Cambridge, UK). Used primary antibodies were: anti-GAPDH (clone 14C10; Cell signaling, #2118), anti-RRM1 (clone T-16; Santa Cruz, sc-11733), anti-RRM2 (clone N-18; Santa Cruz, sc-10844).

### *Fancg*−/− mice

A *Fancg*^*−/−*^ mouse model was generated wherein C57BL/6 J zygotes were injected with in vitro transcribed Cas9 mRNA and a px330 plasmid encoding two guide RNAs (gRNAs) targeting exon 1 and 14 of *Fancg*. Mice were maintained on a pure C57BL6/J background. Eight to 16-week-old mice were used for experiments and were maintained in individually ventilated cages (Innovive) under specific pathogen-free conditions. Experiments were approved by an independent animal ethics committee of the Netherlands Cancer Institute (Amsterdam, Netherlands) (DEC number 14053) and executed according to Dutch and European guidelines.

### In vivo toxicity experiments

WT or *Fancg−/−* mice were administered once with PBS (intraperitoneally (IP), gemcitabine (120 mg/kg, IP), or cisplatin (0.8 mg/kg, intravenous (IV)). Blood smears, tissues, and femur-tibia were harvested on either day 2 or day 7 post-treatment. The femur-tibia was used to isolate bone marrow for flow cytometry analysis as discussed below. Histopathology was performed on blood smears and the tissues as discussed under the section ‘histopathology’.

### Immune fluorescence of bone marrow (BM) cells

Performed as previously described [25].

### Histopathology

The heart, lungs, spleen, thymus, sternum, small intestine, and testis/ovary were collected and fixed in ethanol-glacial acetic acid-formalin (EAF). Upon paraffin embedment, 4 µm sections were made and stained with hematoxylin and eosin (H&E). The sections were reviewed with a Zeiss Axioskop2 Plus microscope (Carl Zeiss Microscopy, Jena, Germany), and images were captured with a Zeiss AxioCam HRc digital camera and processed with AxioVision 4 software (both from Carl Zeiss Vision, München, Germany). The scale bars were set at 50 μm. Blood smears were stained with Wright–Giemsa stains.

### Software and statistical analysis

Statistical analysis, AUC calculations, and visual representation of graphs were performed using GraphPad version 8. R version 3.6 ggplot was used for heatmap visualization. The viability scores as presented in Fig. 1 were obtained as described [14]. Assays were performed in triplicate and standard deviations are shown. For all siRNA viability data shown, the median was calculated relative to the negative (siCONTROL#2) and positive (si*UBB*) tranfection controls, to correct for both toxicity and transfection efficacy.

### Supplementary information

Table S1

Table S2

Figure S1

Figure S2

Figure S3

Figure S4

Figure S5

Supplementary legends

## Data Availability

All original data and reagents can be made available upon request and under a material transfer agreement for privacy protection.
