# Supplementary material for: Gemcitabine as chemotherapy of head and neck cancer in Fanconi anemia patients
Source: Oncogenesis. 2024 Jul 11;13(1):26. doi: 10.1038/s41389-024-00525-2 (PMC11239817; doi:10.1038/s41389-024-00525-2)
Supplement: Supplementary file 1 — Table S1 [file 41389_2024_525_MOESM1_ESM.docx]

|  | | **Supplementary Table S1: Corrected values of validation transfections with si*RRM1* and si*RRM2* per cell line** | | | | | | |
| --- | --- | --- | --- | --- | --- | --- | --- | --- |
|  |  | |  |  |  |  |  |  |
|  | **Cell lines*** | |  | **si*RRM1*** | ***SD*** | **si*RRM2*** | ***SD*** | **Mean of both genes** |
| SENSITIVE | **VU-SCC-094** | | HPV-neg | 0.10 | *0.01* | 0.08 | *0.01* | 0.09 |
|  | **VU-preSCC-M3** | | Pre-HN | 0.09 | *0.05* | 0.13 | *0.04* | 0.11 |
|  | **UM-SCC-22A** | | HPV-neg | 0.13 | *0.05* | 0.09 | *0.01* | 0.11 |
|  | **VU-SCC-017** | | HPV-neg | 0.26 | *0.03* | 0.06 | *0.02* | 0.16 |
|  | **VU-SCC-096** | | HPV-neg | 0.23 | *0.01* | 0.12 | *0.00* | 0.17 |
|  | **CCH-FAHNSCC-2** | | FA-HNSCC | 0.28 | *0.04* | 0.08 | *0.04* | 0.18 |
|  | **UT-SCC-45** | | HPV-pos | 0.27 | *0.06* | 0.10 | *0.02* | 0.18 |
|  | **VU-SCC-120** | | HPV-neg | 0.31 | *0.08* | 0.17 | *0.04* | 0.24 |
|  | **UM-SCC-11B** | | HPV-neg | 0.35 | *0.01* | 0.14 | *0.02* | 0.25 |
|  | **VU-SCC-1604** | | FA-HNSCC | 0.21 | *0.03* | 0.32 | *0.06* | 0.27 |
|  | **VU-SCC-1131** | | FA-HNSCC | 0.20 | *0.03* | 0.34 | *0.09* | 0.27 |
|  | **VU-SCC-1365** | | FA-HNSCC | 0.35 | *0.02* | 0.23 | *0.03* | 0.29 |
| RESISTANT | **FaDu** | | HPV-neg | 0.59 | *0.09* | 0.13 | *0.02* | 0.36 |
|  | **UM-SCC-104** | | HPV-pos | 0.47 | *0.02* | 0.30 | *0.03* | 0.39 |
|  | **D34** | | Pre-HN | 0.33 | *0.05* | 0.44 | *0.05* | 0.39 |
|  | **VU-SCC-078** | | HPV-neg | 0.48 | *0.02* | 0.30 | *0.02* | 0.39 |
|  | **UM-SCC-038** | | HPV-neg | 0.49 | *0.03* | 0.40 | *0.04* | 0.44 |
|  | **VU-SCC-OE** | | HPV-neg | 0.33 | *0.13* | 0.58 | *0.28* | 0.46 |
|  | **UM-SCC-47** | | HPV-pos | 0.57 | *0.04* | 0.55 | *0.03* | 0.56 |
|  | **UM-SCC-6** | | HPV-neg | 0.93 | *0.04* | 0.52 | *0.05* | 0.73 |
|  | **D20** | | Pre-HN | 0.79 | *0.05* | 0.96 | *0.03* | 0.87 |
|  |  | |  |  |  |  |  |  |
|  | | **Cell line order is based on mean viability value of both genes* | | | | | |  |
