# Supplementary material for: Gemcitabine as chemotherapy of head and neck cancer in Fanconi anemia patients
Source: Oncogenesis. 2024 Jul 11;13(1):26. doi: 10.1038/s41389-024-00525-2 (PMC11239817; doi:10.1038/s41389-024-00525-2)
Supplement: Supplementary file 2 — Table S2 [file 41389_2024_525_MOESM2_ESM.docx]

| **Supplementary Table S2: siRNA materials used** | | |  |
| --- | --- | --- | --- |
|  |  |  |  |
|  | **Item** | **Supplier** | **Cat. No.** |
| **siRNAs** | **siCONTOL #2** | GE Healthcare Dharmacon | D-001206-14 |
|  | **si*UBB*** | GE Healthcare Dharmacon | M-013382-01 |
|  | **si*RRM1* pool** | GE Healthcare Dharmacon | *Four single siRNAs pooled* |
|  | **si*RRM1* #1** | GE Healthcare Dharmacon | D-004270-01 |
|  | **si*RRM1* #2** | GE Healthcare Dharmacon | D-004270-02 |
|  | **si*RRM1* #3** | GE Healthcare Dharmacon | D-004270-03 |
|  | **si*RRM1* #4** | GE Healthcare Dharmacon | D-004270-04 |
|  | **si*RRM2* pool** | GE Healthcare Dharmacon | *Four single siRNAs pooled* |
|  | **si*RRM2* #1** | GE Healthcare Dharmacon | D-010379-01 |
|  | **si*RRM2* #2** | GE Healthcare Dharmacon | D-010379-03 |
|  | **si*RRM2* #3** | GE Healthcare Dharmacon | D-010379-05 |
|  | **si*RRM2* #4** | GE Healthcare Dharmacon | D-010379-26 |
|  | **si*RRM2B* pool** | GE Healthcare Dharmacon | M-010575-00-0005 |
