# Supplementary figures and images for: Gemcitabine as chemotherapy of head and neck cancer in Fanconi anemia patients
Source: Oncogenesis. 2024 Jul 11;13(1):26. doi: 10.1038/s41389-024-00525-2 (PMC11239817; doi:10.1038/s41389-024-00525-2)

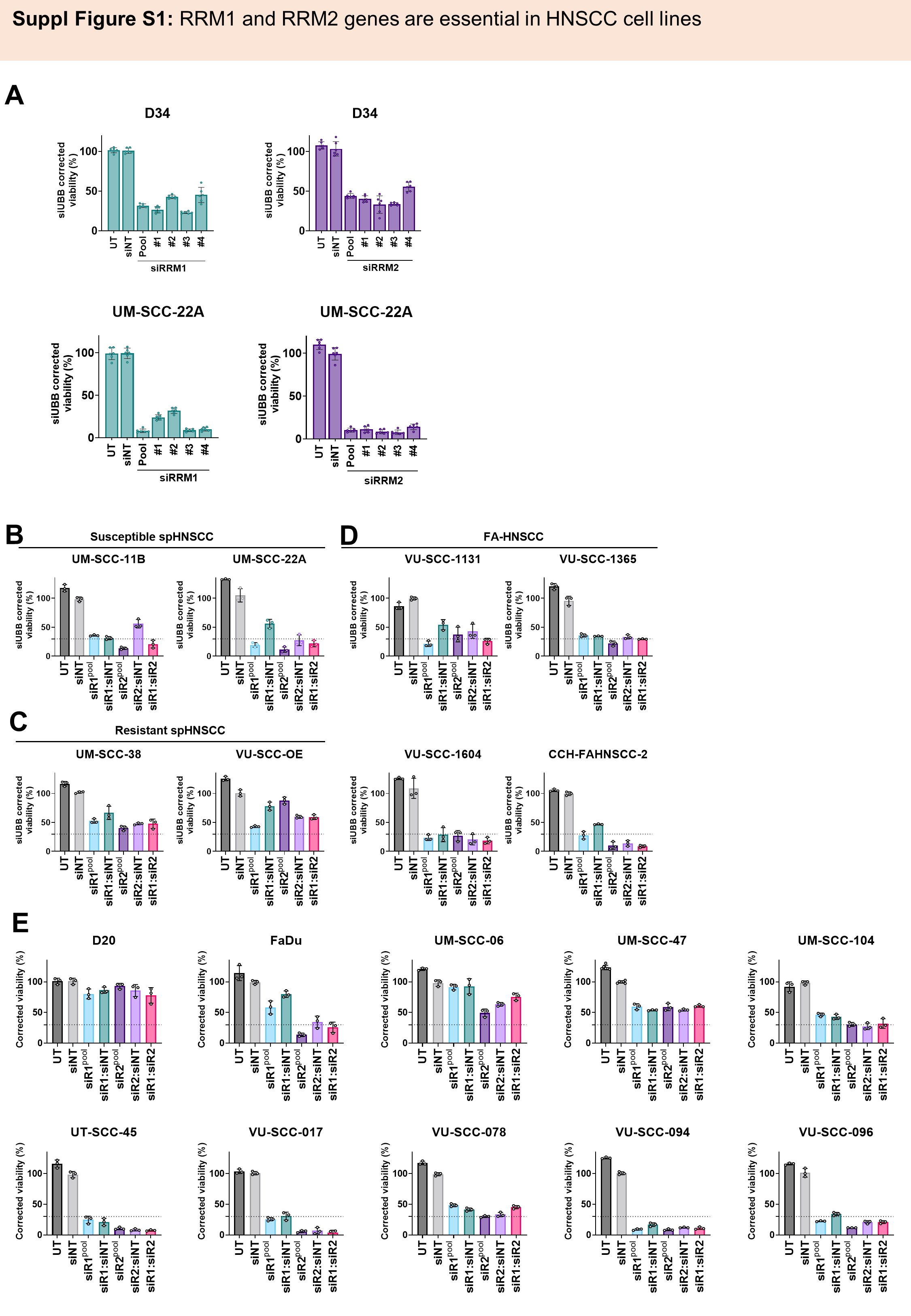

Supplement: Supplementary file 3 — Figure S1 [file 41389_2024_525_MOESM3_ESM.jpg]

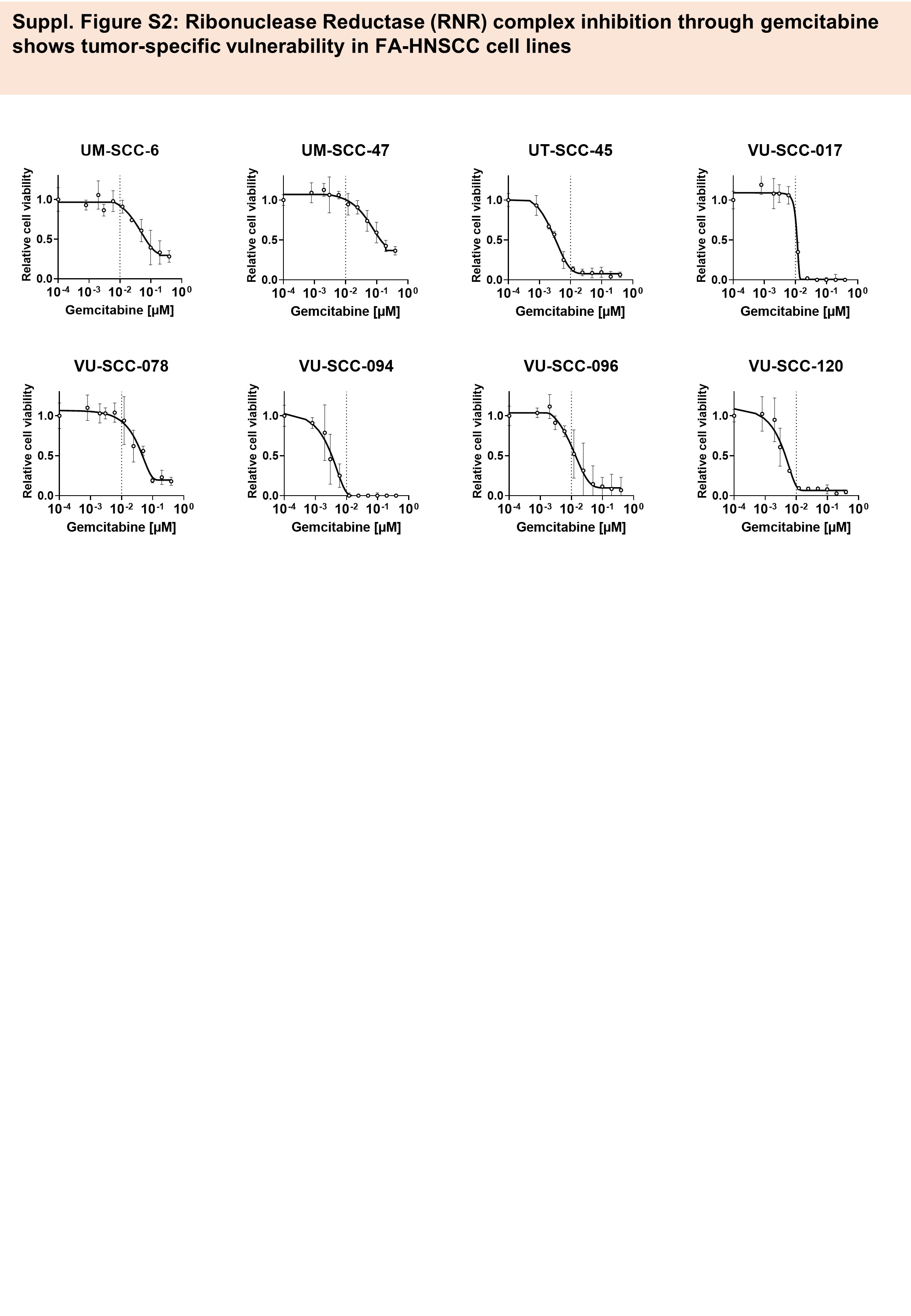

Supplement: Supplementary file 4 — Figure S2 [file 41389_2024_525_MOESM4_ESM.jpg]

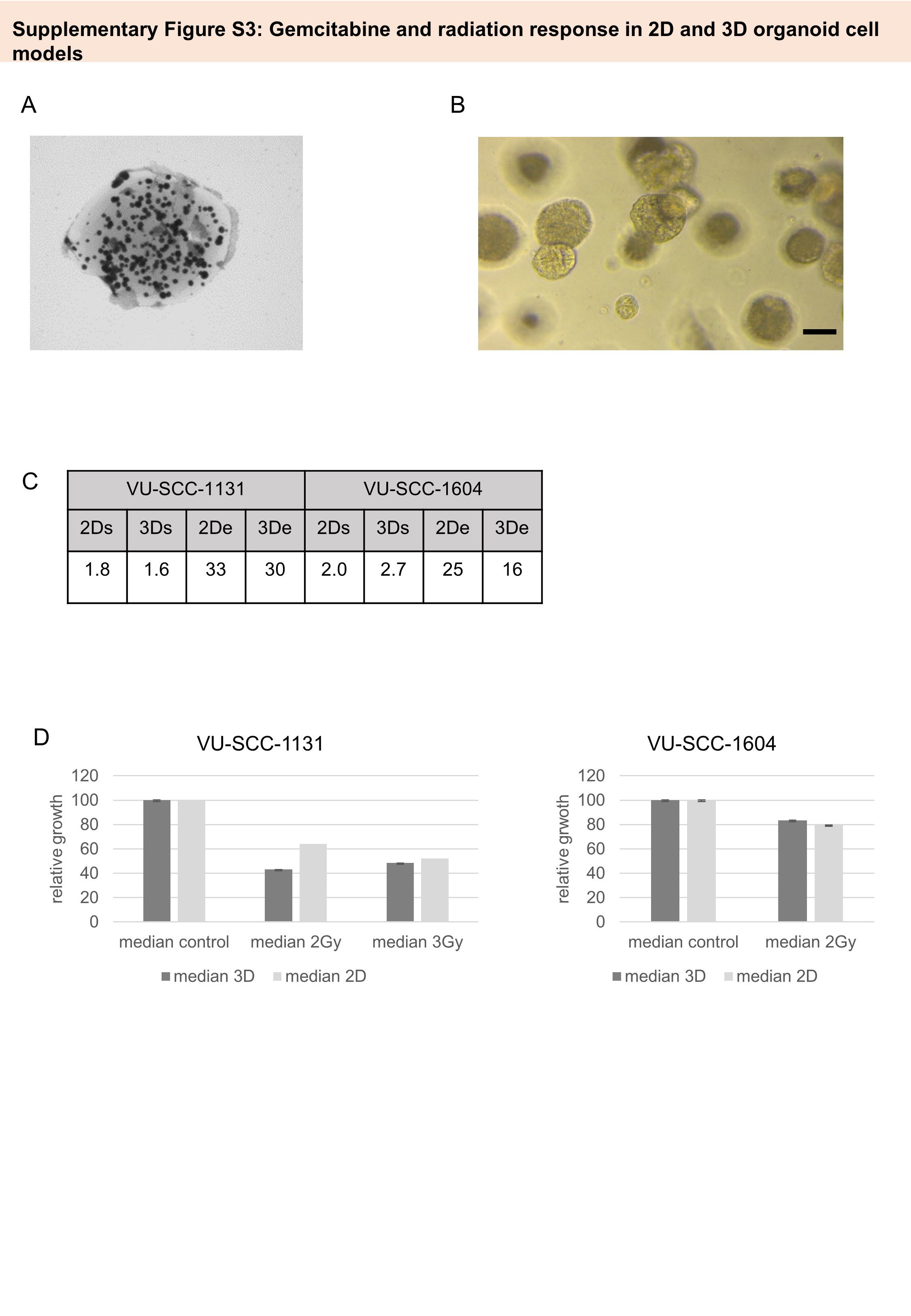

Supplement: Supplementary file 5 — Figure S3 [file 41389_2024_525_MOESM5_ESM.jpg]

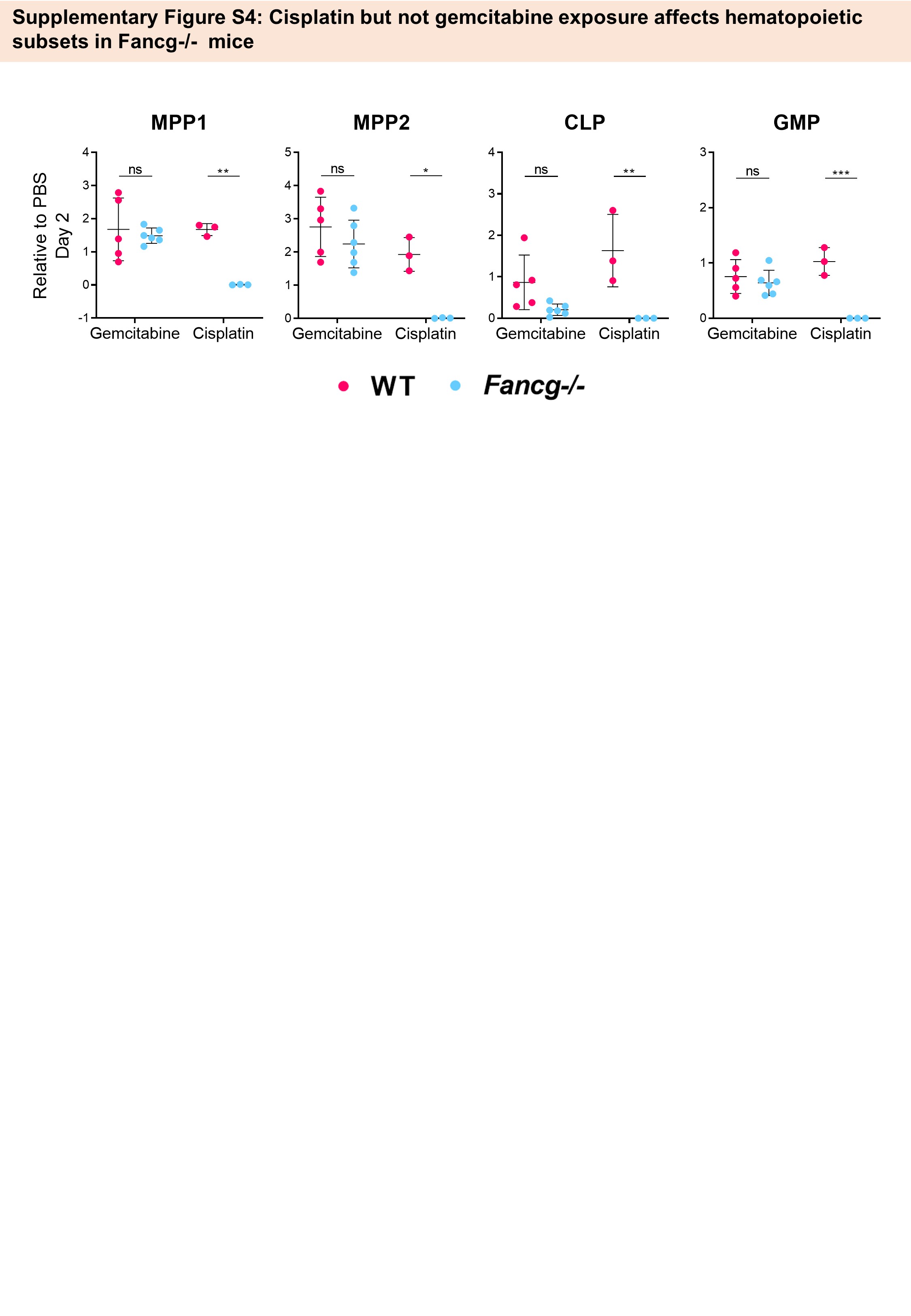

Supplement: Supplementary file 6 — Figure S4 [file 41389_2024_525_MOESM6_ESM.jpg]

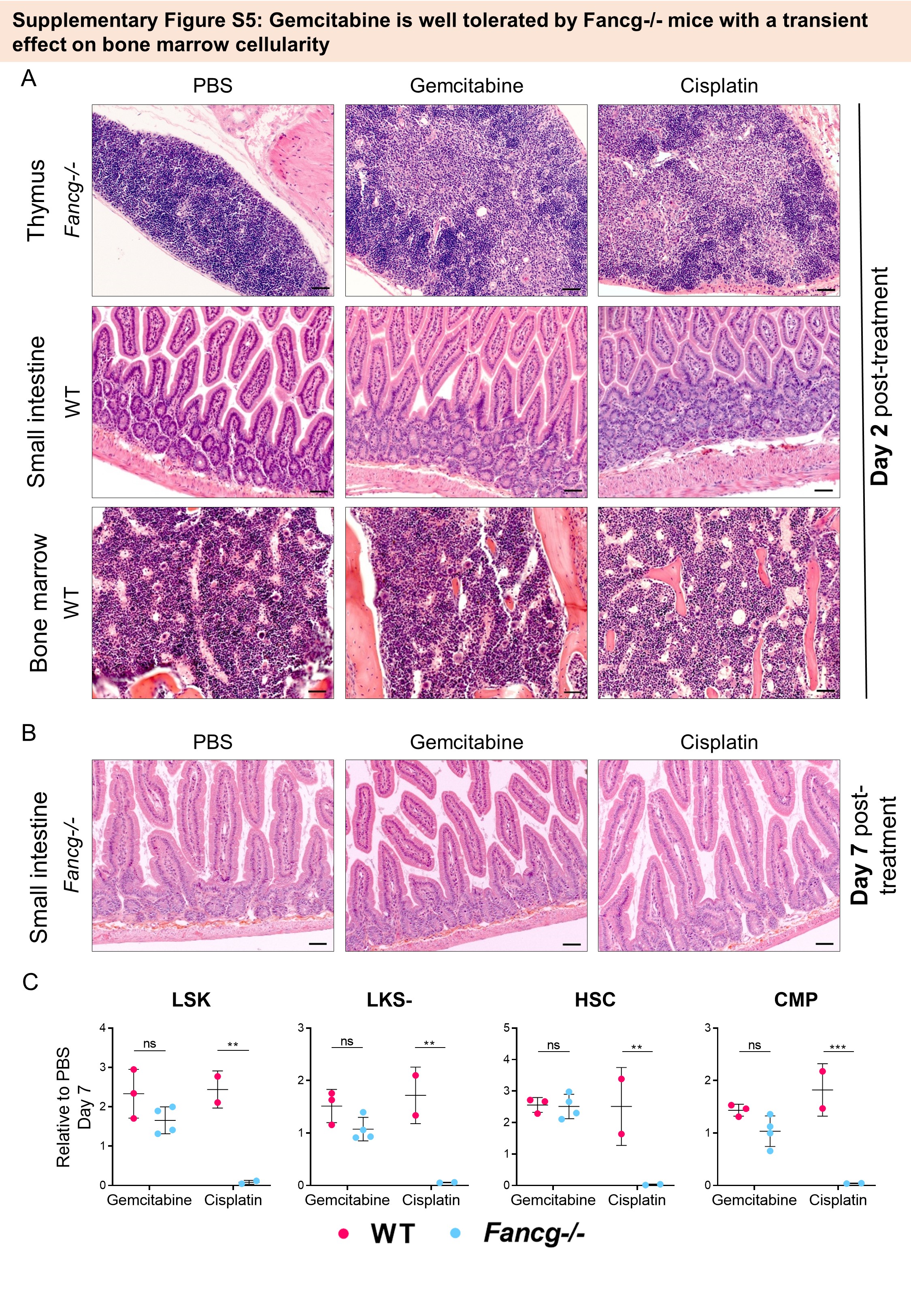

Supplement: Supplementary file 7 — Figure S5 [file 41389_2024_525_MOESM7_ESM.jpg]
