# Supplementary material for: Gemcitabine as chemotherapy of head and neck cancer in Fanconi anemia patients
Source: Oncogenesis. 2024 Jul 11;13(1):26. doi: 10.1038/s41389-024-00525-2 (PMC11239817; doi:10.1038/s41389-024-00525-2)
Supplement: Supplementary file 8 — Supplementary legends [file 41389_2024_525_MOESM8_ESM.docx]

**Suppl. Figure S1: RRM1 and RRM2 genes are essential in HNSCC cell lines**

A) Deconvolution of siRNAs targeting RRM1 (turquoise) and RRM2 (purple) by using the pooled siRNA and the subsequent individual siRNAs (#1-#4) in premalignant oral cells (D34) and HPV-negative cell line UM-SCC-22A. Viability was normalized to siNT and corrected for the transfection efficiency determined by siUBB.

B-E) Combined transfection of siRNAs targeting RRM1 (R1) and RRM2 (R2) with dilution controls using siNT. Viablility was normalized to siNT and corrected for transfection efficiency using siUBB.

**Suppl. Figure S2: Ribonuclease Reductase (RNR) complex inhibition through gemcitabine shows tumor specific vulnerability in Fanconi anemia derived FA-HNSCC cell lines, but not in primary non-transformed oral cells**

Viability of a panel of non-FA-HNSCC cell lines after 72h of gemcitabine exposure.

**Supplementary Figure S3: Gemcitabine and radiation response in 2D and 3D organoid cell models**

A) Macroscopic picture of tumor-like culture models in 5 µl Geltrex matrix, stained with violet blue 5 days after inoculation with 300 cells.

B) Microscopic picture of 3D organoid cultures. Horizontal bar is 10 µm.

C) Comparison of IC50 values in 2D and 3D cultures with gemcitabine added after 24 hrs (2Ds, 3Ds) or 5 days (2De, 3De).

D) Radiation response in 2D and 3D culture models. Experiments were performed in triplicate and the median values are depicted.

**Supplementary Figure S4: Cisplatin but not gemcitabine exposure affects hematopoietic subsets in Fancg-/- mice**

Relative numbers of different subsets as defined in Figure 3B, analyzed on day 2 post treatment. The graphs indicate fold change of cells relative to PBS-controls. In each graph, the bar represents mean ± sd. WT Gem(n=5), Fancg-/- Gem (n=6), WT CsPt (n=3) and Fancg-/- CsPt (n=3). P values were calculated using two-way ANOVA. *p,0.05, **p<0.01, ***p<0.001, ****p<0.0001.

**Supplementary Figure S5: Gemcitabine is well tolerated by Fancg-/- mice with a transient effect on bone marrow cellularity**

A) Representative H&E sections of thymus isolated from Fancg-/- mice and SI and BM from WT mice, all treated with PBS, 120mg/kg gemcitabine or 0.8mg/kg cisplatin and harvested on day 2 post treatment.

B) Representative H&E sections of the small intestine (SI) isolated from Fancg-/- mice treated with PBS, 120mg/kg gemcitabine or 0.8mg/kg cisplatin and harvested on day 7 post treatment.

C) Relative numbers of different subsets as defined in Figure 3B, analyzed on day 7 post treatment. The graphs indicate fold change of cells relative to PBS-controls. In each graph, the bar represents mean ± sd. WT Gem(n=3), Fancg-/- Gem (n=4), WT CsPt (n=2) and Fancg-/- CsPt (n=2). P values were calculated using two-way ANOVA. *p,0.05, **p<0.01, ***p<0.001, ****p<0.0001.
